# New Antibiotics Against Multidrug-Resistant Gram-Negative Bacteria in Lung Transplantation: Clinical Evidence, Safety, and PK/PD Properties

**DOI:** 10.3389/ti.2026.15264

**Published:** 2026-04-10

**Authors:** Andrea Lombardi, Davide Mangioni, Giulia Viero, Laura Alagna, Giulia Renisi, Paola Saltini, Alessandra Bandera

**Affiliations:** 1 Infectious Diseases Unit, Foundation IRCCS Ca’ Granda Ospedale Maggiore Policlinico, Milan, Italy; 2 Department of Pathophysiology and Transplantation, University of Milan, Milan, Italy

**Keywords:** immunocompromised host, lung, MDRGNB, SOT, therapeutic drug monitoring

## Abstract

Infections caused by multidrug-resistant Gram-negative bacteria (MDR-GNB) and *Pseudomonas aeruginosa* are leading causes of morbidity and mortality after lung transplantation (LuTx). We reviewed the pharmacology, clinical evidence, and safety of five agents potentially active against MDR-GNB in LuTx recipients (LUTR): ceftolozane/tazobactam, ceftazidime/avibactam, meropenem/vaborbactam, imipenem/relebactam, and cefiderocol. Literature from the last 10 years was reviewed for data on activity spectrum, efficacy in LUTR and adverse events. Ceftolozane/tazobactam and ceftazidime/avibactam were the most studied, providing high cure rates for difficult-to-treat *Pseudomonas* (DTR-PA) and *Klebsiella pneumoniae* carbapenemase (KPC)-producing Enterobacterales, respectively. Meropenem/vaborbactam offers reliable coverage of KPC strains, while imipenem/relebactam is an interesting option for imipenem-non-susceptible *Pseudomonas* spp. Cefiderocol exhibits the broadest *in vitro* spectrum, including metallo-β-lactamase producers. Across agents, pharmacokinetic variability, augmented renal clearance, and extracorporeal support can compromise target attainment; prolonged or continuous infusion is preferred. Collectively, these antibiotics expand the therapeutic armamentarium against MDR-GNB in LUTR, allowing pathogen-directed, toxicity-sparing regimens. Nonetheless, prospective LuTx-focused studies are needed to optimise their use in such a peculiar setting.

## Introduction

Infections due to Gram-negative bacteria represent most clinically relevant infections among lung transplant (LuTx) recipients (LUTR) in the first year after transplantation and involve primarily the respiratory tract [1]. A growing proportion of these infections is caused by multidrug-resistant Gram-negative bacteria (MDRGNB). Infections due to MDRGNB have been associated with poorer clinical outcomes [2]. This has also been verified in the LuTx setting, with in-hospital mortality rates six times higher in LUTR with infections due to MDRGNB compared to non-MDRGNB [3]. However, evidence suggests that employing an antibiotic effective against the MDRGNB bacteria, especially in *Klebsiella pneumoniae* carbapenemase (KPC)-producing strains or Difficult-to-treat *Pseudomonas aeruginosa* (DTR-PA), can counterbalance this excess mortality [4]. Therefore, it appears clear that the new molecules against MDRGNB that have become available in the last few years could improve the outcomes of MDRGNB infections in LUTR.

We consider in this review ceftolozane/tazobactam (C/T), ceftazidime/avibactam (CZA), meropenem/vaborbactam (MVB), imipenem/cilastatin/relebactam (I-R) and cefiderocol (FDC). Their arrival has provided therapeutic opportunities for difficult-to-treat infections, and scientific societies have endorsed their use for several conditions in which MDRGNB are the culprit [5–7].

In this review, we analyse these new molecules from the perspective of LuTx, focusing on their activity spectrum, safety profile and pharmacokinetic/pharmacodynamic (PK/PD) properties, including therapeutic drug monitoring (TDM). Table 1 provides an overview of common MDRGNB resistance mechanisms and profiles, along with the corresponding activity of the molecules discussed in this review.

**TABLE 1:** Activity spectrum of recently approved antibiotics against multidrug-resistant Gram-negative bacteria.

Antibiotic (year of approval by EMA)	ESBL	KPC	MBL	Amp-C	Oxa-48	P.aer-DTR^a^	CRAb	*S. maltophilia*
Ceftolozane/tazobactam (2015)	✓/✗	✗	✗	✓	✗	✓	✗	✗
Ceftazidime/avibactam (2016)	✓	✓	✗	✓	✓	✓/✗	✗	✗
Meropenem/vaborbactam (2018)	✓	✓	✗	✓	✗	✗	✗	✗
Imipenem/relebactam (2020)	✓	✓	✗	✓	✗	✓	✗	✗
Cefiderocol (2020)	✓	✓	✓	✓	✓	✓	✓	✓

ESBL: extended-spectrum β-lactamases; KPC: *Klebsiella pneumoniae* carbapenemase; MBL: metallo-β-lactamase; Amp-C: AmpC β-lactamases; OXA-48: OXA-48, carbapenemase; P.aer-DTR: difficult-to-treat *Pseudomonas aeruginosa*; CRAb: carbapenem-resistant *Acinetobacter baumannii*.

^a^ Non-MBLs producing.

## Ceftolozane/Tazobactam

### Activity Spectrum

Ceftolozane/tazobactam (C/T) is the combination of ceftolozane, a fifth-generation cephalosporin, with the β-lactamase inhibitor tazobactam. Due to its ability to evade key resistance mechanisms of *P. aeruginosa*, C/T exhibits potent activity against multidrug-resistant (MDR) and extensively drug-resistant (XDR) *P. aeruginosa,* including carbapenem-resistant *P. aeruginosa* (CRPA) [8]. Additionally, it is partially effective against extended-spectrum β-lactamase (ESBL)-producing Enterobacterales*,* showing a preserved susceptibility in ∼85% of isolates [9, 10]. However, it lacks efficacy against carbapenem-resistant Enterobacterales (CRE). C/T present no efficacy against MDRGNB producing carbapenemases, however, it could retains some sensibility against CRE with other resistance mechanism such as porin-mutation or increase membrane efflux [11, 12]. C/T is approved for the treatment of complicated urinary tract infections (cUTI), complicated intra-abdominal infections (cIAI), and ventilator-associated bacterial pneumonia (VABP). According to both IDSA and ESCMID guidelines, C/T is considered the agent of choice for treating infections caused by difficult-to-treat (DTR) *P. aeruginosa* [5, 7]. While the IDSA guidelines place C/T on par with CZA and I-R as first-line options, the ESCMID guidelines identify it as the preferred first-line agent. This indication was set as a consequence of the CACTUS study, which has shown a C/T superiority in the treatment of DTR- *P. aeruginosa* pneumonia, compared to CZA (63% versus 51% in clinical success) [13]. Moreover, CZA has been reported to be associated with higher rates of resistance development; thus, in DTR-*P. aeruginosa* isolates susceptible to both C/T and CZA, C/T may represent a more appropriate therapeutic option in order to reduce the antibiotic selective pressure [14].

For patients with normal renal function, the approved dosage is 1.5 g administered in 1 h every 8 h for cUTI and cIAI, and 3 g administered in 1 h every 8 h for VABP.

### Evidence in the Clinical and LuTx Setting

A 2023 study on 163 *P. aeruginosa* isolates obtained from patients with cystic fibrosis (CF) and LuTx, reported that 81.6% of them were susceptible to C/T [15]. Among MDR and XDR isolates, 88.3% and 28.1%, respectively, were susceptible to C/T. Similarly, Pfaller et al. analysed the susceptibility of 17,315 MDRGNB isolates [16]. They found that *P. aeruginosa* susceptibility was similar between patients >65 years and immunocompromised hosts (ICH), but notably lower in Intensive care unit (ICU) patients: 96.5% vs. 99.1%/99.2% (ICH/>65 years, respectively) in samples from the US, and 80.1% vs. 93.4%/92.5% in samples from Europe.

Clinical evidence on the use of C/T in LUTR remains limited, summarised in Table 2. In a recent French prospective cohort study involving 63 CF patients, of whom 19% (12/63) were LUTR, with *P. aeruginosa* lower respiratory tract infections (LRTI) treated with C/T, 89.3% of the strains were susceptible to C/T. The median treatment duration with C/T was 15 days, and clinical improvement was observed in 88.9% of patients [17].

**TABLE 2:** Overview of real-life studies describing ceftolozane/tazobactam use among LuTx recipients.

Author, year	Country	Study design	Pathogen	Infection type	Main results	AE
Burgel, [17]	France	Prospective cohort study on 63 patients with CF of whom 12 (19%) where LuTx recipients	*Pseudomonas aeruginosa* (97.6%) Escherichia *coli*, *Citrobacter koseri*, *Proteus mirabilis*, and *Serratia marcescens*	LRTI	- C/T susceptibility: 89.3% - Clinical improvement: 88.9% - Mean FEV_1_ improved from 1.33 L to 1.47 L before and after C/T treatment, respectively (p = 0.057)	Two (3.2%) leading to therapy discontinuation (pruritus, skin rash), but no new safety concerns identified
Hart, [18]	United States	Retrospective cohort study of 69 immunocompromised hosts of whom 47 (68%) were SOT recipients	MDR *Pseudomonas aeruginosa*	LRTI (57%) and wound infection (12%)	- 30-day all all-cause mortality: 19% (13/69) - Clinical cure: 68% (47/69) [higher in patients with respiratory tract infections who received 3-g regimens vs. 1.5-g regimens (75% vs. 30%).]	No data provided
Haidal, [19]	United States	Retrospective cohort study of 21 patients whom 7 (33%) were LuTx	MDR *Pseudomonas aeruginosa*	86% LRTI, less common BSI, cIAI, or cUTI	- 30-day all-cause mortality: 10% (2/21) - 30-day attributable mortality 5% (1/21) - C/T failure rate: 29% (6/21) - Resistance to C/T: 14% (3%)	One leading to therapy discontinuation (skin rash); two patients developed thrombocytopenia while on linezolid and linezolid + valganciclovir
Amore [20]	Italy	Case series of 7 LuTx recipients, of whom 4 (57%) were treated with C/T	MDR *Pseudomonas aeruginosa*	LRTI	- Mortality: 1/7 (primary graft dysfunction)	No severe AE occurred
Stokem [21]	United States	Case report of one LuTx recipients with CF	MDR *Pseudomonas aeruginosa*	Pulmonary exacerbation	- Clinical and laboratory improvement - Adequate pharmacokinetic levels	No AE occurred
Escolà-Vergè [22]	Spain	Retrospective cohort study of 38 patients treated with C/T of whom 10 (26.3%) were LuTx	XDR *Pseudomonas aeruginosa*	LRTI, ABSSSI, UTI (not clear the site of infection in LuTx)	- Clinical and laboratory improvement - Adequate pharmacokinetic levels - C/T resistence - All-cause mortality 5/38	No severe AE; CD enteritis (1/38)

AE: adverse event; CF: cystic fibrosis; LRTI: lower respiratory tract infection; C/T: ceftolozane/tazobactam; FEV_1_: forced expiratory volume in 1 s; SOT: solid organ transplant; MDR: multi-drug-resistant; LuTx: lung-transplant recipient; BSI: bloodstream infection; cIAI: complicated intrabdominal infection; cUTI: complicated urinary tract infection.

Another multicentre retrospective cohort study of 69 ICH patients, of whom 68% with a history of solid organ transplant (SOT), assessed the outcomes of C/T use in different infections due to MDR *P. aeruginosa*. The most frequent infection sites were the LRTI (57%). The mean length of C/T therapy was 13 ± 10.8 days. The all-cause 30-day mortality rate among the entire cohort was 19% (13/69), while clinical cure was achieved in 68% (47/69) of patients. This rate was higher in patients with LRTI infections who received 3-g regimens compared to those who received 1.5-g regimens (75% vs. 30%) [18].

Haidal et al. conducted a retrospective study of 21 patients, among whom 7 were LUTR, treated with C/T for MDR *P. aeruginosa* infections. Most patients (18/21, 86%) had LRTI. The 30-day all-cause and attributable mortality were 10% (2/21) and 5% (1/21), respectively, and the C/T failure rate was 29% (6/21). Resistance to C/T emerged in three patients (14%), primarily associated with *de novo* mutations. Overexpression and mutations of AmpC were identified as potential mechanisms underlying this resistance [19].

### Adverse Events and Limitations

Data regarding the concomitant use of C/T and immunosuppressive agents in SOT recipients remain scarce. Ceftolozane is unlikely to cause clinically relevant drug–drug interactions. Conversely, tazobactam is a substrate of organic anion transporters 1 and 3, and coadministration of inhibitors of these transporters may elevate tazobactam plasma concentrations, warranting cautious monitoring. However, as shown by real-world data, C/T is generally well-tolerated, and the most frequently reported AEs are nausea, vomiting, and diarrhoea.

### Key Messages

Clinical data regarding the use of C/T in the LuTx setting are still scarce. C/T is considered the first-line agent for MDR *P. aeruginosa*, which, in the LuTx setting, is among the most frequently isolated pathogens [23, 24]. In this context, the use of C/T may represent a valuable therapeutic option.

## Ceftazidime/Avibactam

### Activity Spectrum

Ceftazidime/avibactam (CZA) combines the third-generation anti-pseudomonal cephalosporin ceftazidime with the novel non-β-lactam BLI avibactam, restoring ceftazidime’s *in vitro* activity against Ambler class A, class C, and specific class D β-lactamases [25]. However, it remains ineffective against metallo-β-lactamase (MBLs). The primary function of this agent is to treat CRE. To address infections caused by MBL-producing bacteria, CZA is co-administered with aztreonam, taking advantage of their synergistic activity [26]. CZA is currently approved for the treatment of cIAI, UTI, and HBAP/VBAP.

For patients with normal renal function, the recommended dosage is 2.5 g administered in 2 h every 8 h.

### Evidence in the Clinical and LuTx Setting

The most significant data regarding the use of CZA in LuTx are described in Table 3.

**TABLE 3:** Overview of real-life studies describing ceftazidime/avibactam use among LuTx recipients.

Author, year	Country	Study design	Pathogen	Infection type	Main results	AE
Chen, [26]	China	Retrospective study on 15 LUTR	CRPA	Not reported	- 14-day mortality: 6.7% - 30-day mortality: 13.3% - Clinical cure: 53.3% - Microbiological cure: 60.0% - Recurrence: 3/15 (20%)	Not reported
Chen, [27]	China-Japan	Retrospective study on 10 LUTR	XDR-GNB (CRKP, CRPA)	PN, BSI, cIAI,	- 30-day mortality: 100% - 90-day mortality: 90% - Relapse of CRKP or CRPA: 5/10 patients (50%) - Microbiological cure: 90.0% - Clinical response: WBC and PCT at 7 and 14 days significantly dropped (p < 0.05) PaO2/FiO2 ratio significantly dropped (p < 0.05)	No severe AE occurred (2 patients experienced increase of urea and creatinine levels)
Amore, [20]	Italy	Case series of 7 LuTx recipients, of whom 4 (57%) were treated with C/T	*K. pneumoniae* MDR (1 coinfection with *P. aeruginosa*)	LRTI	- Mortality: 1/7 (primary graft dysfunction)	No severe AE occurred
Peres-Nadales, [28]	Spain	Retrospective cohort study of 149 SOT, of whom 6 (4%) were LUTR	CPKP	BSI	- CZA treated patients had higher 14-day clinical success: 80.7% vs. 60.6% of BAT (p = 0.011) - CZA treated patients had higher 30-day clinical success: 83.1% vs. 60.6% of BAT (p = 0.004) - CZA treated patients had lower 30-day mortality: 13.3% vs. 27.3% of BAT (p = 0.053)	Not reported
Daccò, [29]	Italy	Case report	*Burkholderia multivorans*	BSI and brain abscesses	- Successful treatment	Not reported
Canton Bulnes, [30]	Spain	Case report	*Burkholderia cepacia* complex	BSI, LRTI	- Successful treatment	Not reported

AE: adverse event; LUTR: lung transplant recipient; CRPA: Carbapenem-resistant *Pseudomonas* aeruginosa; XDR-GNB: Extensively drug-resistant gram-negative bacilli; CPKP: carbapenemase producing *klebsiella pneumoniae*; BSI: bloodstream infection; LRTI: lower respiratory tract infection; cIAI: Complicated intra-abdominal infection; CZA: ceftazidime-avibactam; MDR: multi-drug resistant; BAT: best available therapy; SOT: solid organ transplant; CRE: Carbapenem-resistant Enterobacterales.

A Chinese retrospective observational study on 15 LUTR, investigating the use of CZA in infections caused by CRPA, reported 14-day and 30-day mortality rates of 6.7% and 13.3%, respectively. Moreover, clinical and microbiological cure rates after CZA therapy were 53.3% and 60% [27].

A similar retrospective study conducted on 10 LUTR treated with CZA for carbapenem-resistant *K. pneumoniae* (CRKP) and CRPA infections showed 30-day and 90-day survival rates of 100% and 90%, respectively. However, recurrent CRKP and CRPA infection did occur in 50% of patients [31].

An international, retrospective cohort study evaluated the efficacy of CZA compared with best available therapy (BAT) in a cohort of 149 SOT recipients with BSI caused by CRKP. LUTR accounted for 4% of the overall SOT population, and among the 83 patients treated with CZA, two were LUTR. Treatment with CZA was associated with a significantly higher rate of clinical success at day 14 compared to BAT (80.7% vs. 60.6%). A similar pattern was observed for clinical success at day 30, with statistically significant differences favouring CZA [27].

Notably, CZA treatment was associated with improved survival outcomes in the CAVICOR study, which represents the largest cohort to date investigating the impact of CZA on mortality in infections caused by CRE. However, only 45 out of 339 patients (13.2%) included in the analysis were SOT recipients, and no stratification by type of transplant was provided [32].

### Adverse Events and Limitations

CZA has demonstrated a favourable tolerability profile, with no severe AEs reported in the studies reviewed herein. Only mild AEs were observed. Furthermore, no significant interactions with immunosuppressive therapy were reported.

### Key Messages

Real-world clinical experience with CZA in LUTR, particularly in CRE infections, remains limited. While available data support the efficacy and safety of CZA in treating *P. aeruginosa* infections, evidence specifically about LUTR remains scarce. Further studies are warranted to evaluate the use of CZA in this population. Additionally, close monitoring is advised during treatment, especially for the potential emergence of CZA resistance in *K. pneumoniae* producing KPC-2 and KPC-33 [33, 34].

## Meropenem/Vaborbactam

### Activity Spectrum

Meropenem/Vaborbactam (MVB) combines meropenem with vaborbactam, a novel non-β-lactam BLI.

The primary function of this agent is to treat Enterobacterales that produce KPC enzymes, including those harbouring KPC genes that confer resistance to CZA [35, 36]. In a comparative analysis involving clinical isolates of KPC-positive Enterobacterales, MVB showed more potent *in vitro* activity compared to other drugs alone [37]. Moreover, MVB demonstrated the highest susceptibility rates against the majority of MDRGNB in a surveillance study that included patients with HBAP [38].

However, vaborbactam does not inhibit Ambler classes B or D carbapenemases. MVB’s activity against other DTR-Gram-negative varies and its activity against *P. aeruginosa*, *Acinetobacter* spp., is generally comparable to that of MEM alone [39].

For patients with normal renal function, the recommended dosage is 2/2 g administered in 3 h every 8 h.

### Evidence in the Clinical and LuTx Setting

Two phase 3 clinical trials have evaluated the efficacy and safety of MVB: the TANGO I trial and the TANGO II trial [40, 41].

The latter is an RCT evaluating the efficacy and safety of MVB versus BAT in adults with CRE infections. Bacteraemia was the more relevant infection (46.8%) while HABAP/VABP was found in 10.6% of patients. ICH, including two SOT recipients, represented 32% of the total cohort. Considering the characteristics of the infections in the population with microbiological confirmation, the trial showed similar mortality rate after 28 days for patients treated with MVB for HABAP/VABP and bacteraemia compared to with BAT (22% vs. 44%, p = NS).

In another retrospective multicentre study, describing clinical characteristics and outcomes of 126 patients treated with MVB for MDRGNB infections, the most common infections were LRTI (38.1%), and the most common isolated pathogens were CRE (78.6%). Thirty-day mortality occurred in 18.3% of patients (n = 23), but only half of these patients received an appropriate dose of medication based on their renal function. Outcomes were similar between patients with CRE and *Pseudomonas* spp. isolates [42].

Lastly, in a retrospective study comparing the efficacy of MVB (n = 26) with CZA (n = 105) in patients with CRE infections, the clinical success rate was similar in both groups, with around half of the patients treated with MVB having an LRTI (n = 12) [41].

### Adverse Events and Limitations

In both the TANGO I and TANGO II trials, patients receiving MVB experienced fewer side effects than those receiving other treatments. In the TANGO II trial, AEs associated with MVB included diarrhoea, anaemia and hypokalaemia. Interestingly, patients receiving MVB treatment experienced a lower incidence of renal failure than those receiving BAT [40, 41].

In a study comparing the efficacy of MVB and CZA, rates of AEs were similar between the CZA group and the MVB group (34.3% versus 23.1%, respectively; p = 0.27). Nephrotoxicity was the most frequent AE, with rates of 29.2% and 14.3% in the CZA and MVB groups, respectively (p = 0.16).

### Key Messages

Considering its broad spectrum of activity and good lung penetration, MVB could be a promising option for LUTR with infections caused by KPC-producing CRE. The use of an adequate dose adjusted to renal function will be a future challenge in using this molecule, to ensure correct drug exposure and minimise AEs and the development of resistance.

## Imipenem/Relebactam

### Activity Spectrum

Imipenem/Relebactam (I-R) combines imipenem with relebactam, a novel BLI without direct antimicrobial activity, but providing reliable inhibition of many Ambler class A and class C [43] β-lactamases, as well as *Pseudomonas*-derived cephalosporinase [43]. Relebactam is not active against MBLs or class D oxacillinases [44].

The activity of I-R is similar to CZA against CRPA. In addition, in a small percentage of cases, I-R also showed activity against those bacteria that had developed resistance to C/T and CZA [45]. It remains ineffective against *A. baumannii* and *Stenotrophomonas maltophilia* and exhibits limited activity against OXA-48-like enzymes [46]. Some data have highlighted the emergence of I-R resistance during this treatment in patients with *P. aeruginosa* HABAP/HAVAP previously exposed to other cephalosporins. This mechanism could be due to increased expression or structural changes in the MexAB-OprM and MexEF-OprN efflux pumps [47, 48].

I-R against KPC-producing Enterobacterales demonstrated good *in vitro* activity. Different *in vitro* strain analyses reported a 98% susceptibility rate for *K. pneumoniae* producing KPC. However, a pooled estimation of around 280,000 isolates revealed an I-R resistance prevalence rate of approximately 14.6% (95% CI, 0.116%–0.182%), with rates exceeding 50% observed in many countries worldwide [49].

For patients with normal renal function, the recommended dosage is 1.25 g administered in 30′ every 6 h.

### Evidence in the Clinical and LuTx Setting

Clinical data on I-R use among LuTx patients is lacking. Safety and efficacy of I-R among patients with VAP or HAP are studied in two Phase 3 non-inferiority trials (RESTORE-IMI-1 and RESTORE-IMI-2).

In the first trial, I-R (n = 21) was compared to colistin plus IMP treatment (n = 10) in patients with IMP-susceptible bacterial infections. Patients were treated for HABAP/HAVAP (n = 11, 35%), cUTI (n = 16, 52%) or cIAI (n = 4, 13%). Favourable overall responses were achieved in both arms (I-R 71%; colistin + IMP, 70%) [50].

The second trial evaluated I-R (n = 246) versus TZP (n = 267) in patients with HABAP/HAVAP. In this trial, critically ill patients were studied, but ICH were excluded. The most common pathogens were *K. pneumoniae* (25.6%), *P. aeruginosa* (18.9%), *Acinetobacter calcoaceticus-baumannii* complex (15.7%), and *Escherichia coli* (15.5%). I-R was non-inferior to TZP, considering 28-day all-cause mortality (15.9% and 21.3% respectively). Instead, on day 28, all-cause mortality in microbiologically modified intent-to-treat patients with a primary diagnosis of HABAP/HAVAP was lower in the I-R arm than the TZP arm (18.6% vs. 30.8%), and the incidence of relapse/clinical failure was comparable between I-R and TZP (14% vs. 12%). Patients with *P. aeruginosa* infections had a lower clinical response and a higher 28-day mortality rate in the I-R arm. Nevertheless, both treatment arms had comparable microbiological eradication rates at the end of treatment (67% for I-R versus 72% for TZP). This result requires further interpretation as it may be due to differences between the treatment groups that are unrelated to the causative pathogen, given the limited sample size in the I-R group [51].

Another randomised non-inferiority trial compared I-R and TZP for the treatment of HABAP/HAVAP. Again, ICH were excluded from this cohort. The study confirmed that I-R was non-inferior to TZP in terms of 28-day all-cause mortality (11% vs. 5.9%; non-inferiority p = 0.024). It should be noted that mortality was numerically higher in the I-R treatment group, despite non-inferiority being reached [52].

### Adverse Events and Limitations

I-R treatment is generally well tolerated. The most common AEs in registration studies were anaemia (10%), nausea and diarrhoea (8%), and elevated liver enzymes (12%) [50, 51].

Regarding renal toxicity, I-R was associated with a more favourable renal safety profile than colistin-based therapy in RESTORE IMI-1. So far, some drug-drug interactions have been described [53]. Carbapenems (imipenem as well as meropenem) have been linked with an increased risk of seizures, especially with the concomitant administration of certain antiepileptic drugs (e.g., valproic acid), due to a marked decrease in those drugs’ levels. Additionally, concomitant use of ganciclovir requires monitoring due to an increased risk of central nervous system toxicity [54]. These interactions are not associated with the new beta-lactamase inhibitor but are inherent to carbapenems.

### Key Messages

The role of I-R among ICH, particularly LUTR, requires further investigation. However, considering the data on patients with pneumonia and its anti-pseudomonal spectrum, I-R could play a promising role in this setting. Caution must be exercised regarding DDI and the potential for resistance to emerge.

## Cefiderocol

### Activity Spectrum

Cefiderocol (FDC) is a novel catechol-substituted siderophore cephalosporin. FDC can bind extracellular iron and use iron-regulated outer membrane proteins to gain access to bacteria. FDC can overcome resistance mechanisms due to efflux pumps, ubiquitous in MDRGNB such as *P. aeruginosa* [55]. Moreover, FDC’s potent activity against MDRGNB is also related to its high stability against various ESBLs and carbapenemases (IMP-1, VIM-2, NDM-1, KPC-2/3, L1, OXA-23) [56]. Moreover, FDC demonstrated *in vitro* activity against AmpC-overproducing strains, a low affinity for chromosomal AmpC β-lactamases, and a low propensity for temporal induction of AmpC β-lactamases [57].

This translates into a potentially vast activity spectrum against MDRGNB, with data from the SIDERO-WT study showing susceptibility rates to FDC of 99.8%, 99.9% and 96% for clinical isolates of Enterobacterales, *P. aeruginosa* and *A. baumannii*, respectively [58]. A recent systematic review reported slightly less favourable proportions, with susceptibility rates to FDC of 97%, 91.2%, and 96% for Enterobacterales, *P. aeruginosa,* and *A. baumannii*, respectively. Of note, FDC-resistance was significant in NDM-producing Enterobacterales (38.8%, 95% CI 22.6%–58.0%), NDM-producing *A. baumannii* (44.7%, 95% CI 34.5%–55.4%), and CZA-resistant Enterobacterales (36.6%, 95% CI 22.7%–53.1%), suggesting a cautious use against these microorganisms [59].

The recommended dosage is 2 g administered in 3 h every 8 h for patients with normal renal function.

### Evidence in the Clinical and LuTx Setting

A growing bulk of evidence is accumulating regarding the use of FDC in IC hosts, including LUTR (Table 4).

**TABLE 4:** Overview of real-life studies describing cefiderocol use among LuTx recipients.

Author, year	Country	Study design	Pathogen	Infection type	Main results	AE
Persaud, [60]	USA	Case series of 15 LUTR	MDR *P. aeruginosa* (14/15)	LRTI	- 30-day all-cause mortality: 26%; - Microbiological clearance: 9/13	Not reported
Soueges, [61]	France	Multicentre retrospective study including 114 ICH (LUTR 14.9%)	*P. aeruginosa* (56%) of whom VIM producers (11.7%)	LRTI (48.2%), cUTI (14%), cIAI (9.6%)	- 28-day clinical success: 53.3% - 28-day mortality: 37.7% - 28-day relaps: 17.5%	Not reported
Torre-Cisneros, [60]	Spain	Early access program analysis including 261 patients (SOT 12.6%)	*P. aeruginosa* (67%), many CZA/C/T resistant	LRTI (47.9%), cIAI (14.6%), cUTI (14.6%)	- 28-day mortality: 21.5% - Clinical cure: 76% (LRTI)	2.2%: one rash, one leukopenia, one fatal toxic epidermal necrolysis
Lombardi, [62]	Italy	Post-hoc multicentre national analysis including 185 patiens (ICH 45.4%, SOT 6.5%)	*P. aeruginosa,* Enterobacterales*, A. baumannii*	Empirical therapy: sepsis (36/54, 66.7%) Targeted therapy: LRTI (56/131, 42.8%)	- 28-day clinical cure: 81% (*P. aeruginosa*), 77.3% (Enterobacterales), 42% (*Acinetobacter baumannii*); - 30-day all-cause mortality: 40.8%	2%: two rash, one increase liver enzyme values, one status epilepticus

AE: adverse event; LUTR: lung transplant recipient; ICH: immunocompromised host; SOT: solid organ transplant; BSI: bloodstream infection; LRTI: lower respiratory tract infection; cIAI: Complicated intra-abdominal infection; cUTI: complicate urinary tract infection; CZA: ceftazidime-avibactam; C/T: ceftolozane/tazobactam; MDR: multidrug-resistant.

Persaud et al. presented a single-centre, retrospective description of FDC among 15 LUTR. FDC was initiated at a median of 105 days post-transplant, with treatment courses ranging from 1 to 93 days. MDR *P. aeruginosa* was the target pathogen in 13 cases. Of the 15 patients, 14 underwent FDC susceptibility testing, with three yielding an intermediate result, despite no prior exposure to the agent. Overall, 30-day mortality was 26% [60].

In the CEFI-ID study, an analysis of FDC use in 114 ICH adults treated for MDRGNB infections, LUTR constituted 15% (17/114) of the study population. LRTI were the most common infection (55/114, 48.2%), and *P. aeruginosa* (51/114, 56%) was the most common pathogen. At day 28, clinical success was achieved in 53.3% of cases, and overall mortality was 37.7%.

The PERSEUS study, an analysis of the 261 Spanish patients with severe infections due to MDRGNB (excluding *Acinetobacter* spp.) enrolled in the FDC early access program, included a relevant proportion of SOT recipients (34/261, 13%). The most represented pathogen was *P. aeruginosa* (174/261, 66.7%), with 99 (76.7%) isolates resistant to both CZA and C/T. Interestingly, the highest 28-day mortality (27.2%) and the second lowest clinical cure rate (76%) were reported among patients with LRTI, the most common infection site (47.9%), suggesting how infections of this compartment are particularly relevant in shaping the clinical course of patients [61].

Finally, in a post-hoc analysis focused on ICH of the first collected data from the prospective, multicenter national CEFI-SITA study, 84 ICH cases were compared to non-ICH cases. Thirty-day mortality was comparable between ICH and non-ICH (40.8%, 95%CI 27.9%–56.8% vs. 33.3%, 95% CI 22.9–46.9; p = 0.5430). In the multivariable analysis, ICH status and its groups were not associated with higher mortality [63].

### Adverse Events and Limitations

FDC is primarily eliminated unchanged in the urine and is not extensively metabolised by the liver [64]. FDC may cause renal impairment [65]. Therefore, it is necessary to closely monitor renal function [64]. Finally, therapeutic and supratherapeutic doses of FDC had no apparent clinically significant effect on the QTc [66].

In the real-life experiences mentioned above, AEs related to FDC were rare or not reported. In the PERSEUS study, 7/314 patients (2.2%) experienced a suspected drug-related AE during FDC administration, and three patients discontinued the drug [60]. In the CEFI-SITA study, 4/200 patients (2.0%) experienced a suspected AE during FDC administration, and FDC was discontinued in two patients [63].

### Key Messages

FDC is a solid addition to the therapeutic armamentarium against MDRGNB in LUTR, and scientific societies have endorsed its use for some conditions. In the field of LuTx, FDC is likely a valid solution for treating LRTI due to MDR *P. aeruginosa* and an alternative for infections caused by MBL-producing Enterobacterales. Further studies are required to understand the need for combination therapy when used in difficult-to-reach sites, such as the lung or the abdomen, and the emergence of resistance.

## PK/PD of New Molecules

All the antibiotics described in this review belong to the β-lactams class. β-lactams are defined as ‘time-dependent’ antibiotics, indicating that their bactericidal activity is dependent on the proportion of time (T) their unbound concentration (*f*) remains above the minimal inhibitory concentration (MIC) of the bacterial pathogen. The PK/PD index is expressed as *f*T > MIC [67]. β-lactams are hydrophilic molecules with a relatively small volume of distribution and are eliminated by renal clearance. Hence, physiopathological changes that frequently occur in critically-ill patients can greatly affect the PK of β-lactams [67, 68]. For these reasons, while 50% *f*T > MIC is likely enough to obtain standard efficacy of β-lactam antibiotics, in critically ill IC individuals up to 100% *f*T >4-6 x MIC should be ensured for optimal drug exposure and suppression of resistance development [67, 69, 70].

Rando et al. recently published a systematic review specifically focused on pulmonary PK/PD data of novel β-lactams. Overall, probabilities of target attainment rates were reported above 90% using current licensed dosing regimens, although significant heterogeneity was reported between studies, both in terms of clinical population and PK models [71].

When dealing with LUTR with severe infections, major strategies to overcome the PK/PD challenges and optimise β-lactam efficacy include prolonged infusion (PI) and TDM. The duration of β-lactams infusion has been shown to influence their *f*T > MIC, increasing the chances of target attainment. Several experimental and clinical studies support PI (either continuous or extended infusion) of β-lactams in the setting of severe infections [69, 70, 72].

In 2024, Abdul-Aziz et al. conducted a meta-analysis of 18 randomised controlled trials comparing PI versus intermittent infusion of β-lactams in critically ill adults with sepsis/septic shock, involving over 9,000 patients. PI was associated with reduced all-cause 90-day mortality (risk ratio 0.86, 95%CrI 0.72–0.98), reduced risk of ICU-mortality (risk ratio 0.84, 95%CrI 0.70–0.97) and an increase in clinical cure (risk ratio 1.16, 95%CrI 1.07–1.31) [72].

In 2022, the first consensus guidance on the use of β-lactams as PI was published. The panel was unanimously in favour of PI over standard infusion in severely ill adult patients, particularly those with MDRGNB infections [70].

TDM consists of measuring a drug concentration in a specific biological sample to help clinicians achieve the PK/PD target. Yet, implementation of β-lactams TDM into a hospital system requires resources, practical workflow considerations and expertise that need to be considered thoroughly [67, 73]. Patients that would benefit the most from β-lactams TDM are those at risk of sub-therapeutic concentrations due to PK variability (e.g., augmented renal clearance, ECMO or renal replacement therapy) or PD characteristics (e.g., MDR bacteria with high MIC values or deep-seated infection with high bacterial inoculum) [67].

In 2022, Pai Mangalore et al. conducted a systematic review and meta-analysis on TDM-guided dosing in over 1,400 critically ill patients. The TDM group was associated with increased target attainment (risk ratio 1.85, 95%CI 1.08–3.16) and improved clinical cure (risk ratio 1.17, 95%CI 1.04–1.31), microbiological cure (risk ratio 1.14, 95%CI 1.03–1.27), and reduced treatment failure (risk ratio 0.79, 95%CI 0.66–0.94) [74].

On the other hand, the clinical benefit of beta-lactam TDM have been questioned. Evidence, including recent meta-analyses, shows no clear impact of TDM on mortality or clinical cure. In general, most beta-lactams are well tolerated, supporting high-dose regimens, even with mild renal impairment, usually guaranteeing high serum concentrations [75].

In the management of LUTR patients, a unique condition is represented by the perioperative period. Candidates often arrive at transplant with respiratory tract colonisation by MDR bacteria and could require novel β-lactams as antibiotic prophylaxis [62]. Antibiotic underdosing in prophylactic regimens may increase the risk of infection and ultimately, graft failure.

In 2020, Taccone et al. published a single-centre retrospective study on 70 LUTR that received prophylactic therapy with a β-lactam antibiotic and underwent TDM in the early postoperative period. Insufficient drug concentrations were found in 28/70 (40%) patients, significantly associated with CF, younger age and increased creatinine clearance. Interestingly, patients with inadequate drug concentrations during postoperative antibiotic prophylaxis developed MDR acquisition and/or early infection more frequently than those with adequate drug concentrations (22/28, 79% vs. 20/42, 48%; p = 0.01) [76].

Studies assessing the PK/PD target attainment of new β-lactams when employed in transplant prophylaxis are required to evaluate the need for routine TDM in this crucial phase of the patients’ management.

## Conclusion

Infections caused by MDRGNB remain a critical challenge in LUTR. The availability of new antibiotics, supported by growing evidence on their PK/PD profiles, safety, and efficacy, offers valuable therapeutic options. However, clinical experience in the LuTx setting is still limited for several agents, and optimal use often requires individualised dosing strategies, TDM, and a deep understanding of local resistance patterns. Future research should prioritise prospective, SOT-specific trials to define the most effective and safe use of these agents and to guide stewardship in this vulnerable population.

## Glossary

ABC: *Acinetobacter baumannii-calcoaceticus* complex
AE: Adverse event
BAT: Best available therapy
BL: β-lactam
BLI: β-lactamase inhibitor
BSI: Bloodstream infection
CF: Cystic fibrosis
CI: Continuous infusion
cIAI: Complicated intra-abdominal infection
CNS: Central nervous system
CRE: Carbapenem-resistant Enterobacterales
CRKP: Carbapenem-resistant *Klebsiella pneumoniae*
CRPA: Carbapenem-resistant *Pseudomonas aeruginosa*
C/T: Ceftolozane/tazobactam
cUTI: Complicated urinary tract infection
CZA: Ceftazidime/avibactam
DTR: Difficult-to-treat
ECMO: Extracorporeal membrane oxygenation
ESBL: Extended-spectrum β-lactamase
FDC: Cefiderocol
HABAP/HAVAP: Hospital-acquired/ventilator-associated bacterial pneumonia
ICH: Immunocompromised host
ICU: Intensive care unit
IPM: Imipenem
I-R: Imipenem-relebactam
KPC: *Klebsiella pneumoniae* carbapenemase
LRTI: Lower respiratory tract infection
LUTR: Lung transplant recipient
MBL: Metallo-β lactamase
MDR: Multidrug-resistant
MDRGNB: Multidrug-resistant Gram-negative bacteria
MIC: Minimal inhibitory concentration
MVB: Meropenem/vaborbactam
NDM: New Delhi metallo-β-lactamase
OXA: Oxacillinase
PI: Prolonged infusion
PK/PD: Pharmacokinetic/pharmacodynamic
SOT: Solid organ transplant
TDM: Therapeutic drug monitoring
TZP: Piperacillin/tazobactam
VIM: Verona Integron-encoded Metallo-β-lactamase
XDR: Extensively drug-resistant
